# Impact of stretch on sarcomere length variability in isolated fully relaxed rat cardiac myocytes

**DOI:** 10.21203/rs.3.rs-3043911/v1

**Published:** 2023-06-14

**Authors:** Oleg Lookin, Najlae Boulali, Olivier Cazorla, Pieter Tombe

**Affiliations:** Université de Montpellier, INSERM, CNRS, CHU Arnaud de Villeneuve; Université de Montpellier, INSERM, CNRS, CHU Arnaud de Villeneuve; Université de Montpellier, INSERM, CNRS, CHU Arnaud de Villeneuve

**Keywords:** isolated cardiomyocyte, sarcomere, sarcomere length variability, carbon fibers, stretch

## Abstract

The contractility of cardiac muscle is greatly affected by preload via the Frank-Starling Mechanism (FSM). It is based on the preload-dependent activation of sarcomeres – the elementary contractile units in muscle cells. Recent findings show a natural variability in sarcomere length (SL) in resting cardiomyocytes that, moreover, is altered in an actively contracting myocyte. SL variability may contribute to the FSM but it remains unresolved whether the change in the SL variability is regulated by activation process *per se* or simply by changes in cell stretch, i.e. average SL.

To separate the roles of activation and SL, we characterized SL variability in isolated fully relaxed rat ventricular cardiomyocytes (*n* = 12) subjected to a longitudinal stretch with the carbon fiber (CF) technique. Each cell was tested in three states: without CF attachment (control, no preload), with CF attachment without stretch, and with CF attachment and ~ 10% stretch of initial SL. The cells were imaged by transmitted light microscopy to retrieve and analyze individual SL and SL variability off-line by multiple quantitative measures like coefficient of variation or median absolute deviation.

We found that CF attachment without stretch did not affect the extent of SL variability and averaged SL. In stretched myocytes, the averaged SL significantly increased while the SL variability remained unchanged. This result clearly indicates that the non-uniformity of individual SL is not sensitive to the average SL itself in fully relaxed myocytes. We conclude that SL variability *per se* does not contribute to the FSM in the heart.

## INTRODUCTION

Sarcomere length (SL) is the fundamental parameter that controls preload-dependent regulation of muscle contraction [6, 7, 14]. The sarcomeric contractile response in cardiac myocyte non-linearly increases in proportion to its length over the physiological range [5]. The in-series arrangement of sarcomeres in myofibrils, therefore, strongly dictates that their individual lengths, simply due to their mechanical arrangement, induce significant mutual effects to each other during mechanical activation [6].

In resting skeletal and cardiac myocytes/myofibrils, SL heterogeneity may be as high as 20% of the average SL [1, 12, 15, 19, 20, 24, 32], and in one report even more [25]. Moreover, SL variability is increased in the actively contracting cardiomyocyte, i.e. in the systolic phase [17, 21] or during steady state activation in skeletal myocytes [3, 12, 15, 16, 25, 26, 27, 30]. However, it remains unresolved whether SL heterogeneity is governed by the (physiological) activation process, or whether it is caused simply by passive mechanical properties of the sarcomere, and thus can be “preadjusted” simply by prestretch (preloading) of the cell.

In our previous study we have shown that the SL variability in an isolated mechanically unloaded and actively contracting cardiomyocyte is significantly higher in the end-systolic state at lower average SL *vs* the end-diastolic state at higher averaged SL [21]. The aim of the present study was to examine whether a physical change in SL itself directly affects SL variability in the absence of the activation process in a cell. We recorded the sarcomere striation signal in transmission light mode with enhanced contrast and high magnification using a confocal microscopy system. Each cell was imaged in the following states: without carbon fiber (CF) attachment (“unattached”), with CF attachment but without stretch (“attached”), and with stretch by CF (“stretched”). The extent of SL variability in each state was thoroughly characterized by multiple statistical measures, both absolute, such as coefficient of variation, inter-quartile range, median absolute deviation, and relative measures.

## METHODS

All investigations conformed to European Parliament Directive 2010/63/EU and approved by the ethics committee “Comité d’éthique pour l’expérimentation animale Languedoc-Roussillon”.

### Isolated cardiomyocytes

Ventricular cardiomyocytes were isolated by enzymatic digestion as previously described [2]. Briefly, male rats (~ 300 g) received a lethal dose of pentobarbital (50 mg/kg). The heart was quickly cannulated via the aorta and perfused with a HEPES physiological solution at 37°C containing type IV collagenase enzyme (1.25 mg/ml, Worthington). After enzymatic digestion, the external Ca^2+^ concentration was set to 0.1 mM. All subsequent experiments were performed using a modified Tyrode solution (140 mM NaCl, 4 mM KCl, 1 mM MgCl_2_, 20 mM HEPES, 0.1 mM CaCl_2_, and 11 mM glucose, pH 7.4) at room temperature without electrical pacing. All solutions contained 20 mM BDM to prevent spontaneous contractile activity.

### Optical measurements

**Figure 1:**
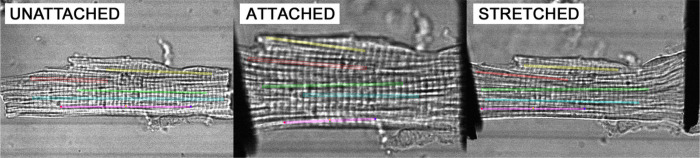
Representative transmission light images of a cardiomyocyte in three states: “unattached” (no attached carbon fibers, CF), “attached” (CF attached but no stretch applied), and “stretched” (myocyte is stretched by CF). Five regions of interest were used in each image to collect sarcomeric data.

The analysis of intracellular variability in sarcomere lengths in isolated cardiomyocytes was performed using transmission light (TL) measurements. Briefly, myocytes were attached on both ends to 7 μm carbon rods using Myotak according to the previously described methods [8]. The CFs were mounted to hydraulic XYZ manipulators that aided in the carbon rod attachment, as well as gentle SL stretch of the attached myocytes. The cells were mounted in a glass bottomed 35mm plastic petri dish placed on top of an inverted microscope (Nikon, TE3000 Eclipse) equipped with a Nikon 60/1.2 water immersion objective. Transmitted light DIC images were obtained with a confocal microscope (Biorad Radiance 2100) using a 488 nm DPSS laser (Melles-Griot), as a 1024×1024 image with ~ 200 nm spatial resolution in the X-Y plane. Representative images of a cardiomyocyte in the three states – unattached to CFs, attached to CFs but not stretched, and stretched by CFs – are shown in Fig. 1. The stretch was approximately 10% of cell length in the resting state without CF attachment (SL stretched from ~ 1.9 μm to ~ 2.2 μm). Measurements were performed on *n* = 12 cardiomyocytes obtained from *n* = 7 hearts.

### Analysis of variability in sarcomere lengths

**Figure 2:**
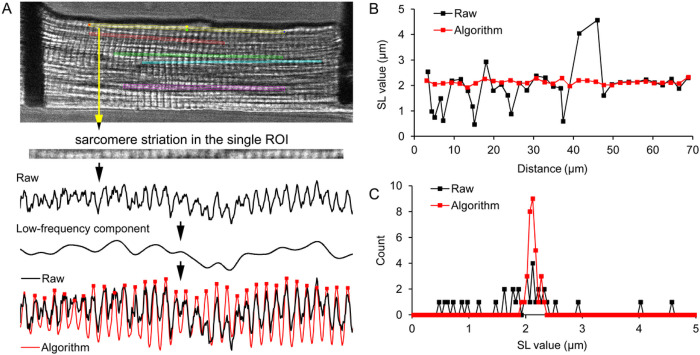
The implementation of the custom-made algorithm designed for calculation of individual sarcomere lengths (SL) using a sarcomere striation pattern. (A) Example of transmitted light image of a cardiomyocyte with 5 regions of interests (ROIs) placed over the whole area of the cell. The sarcomere striation profile is obtained from the selected ROI. The algorithm uses raw data (black curves) as input and first removes non-regular components of the data, then it produces (via several steps, see text for details) the adjusted data with significantly decreased impurities in the regular striation pattern (red curved). The local maxima of the adjusted signal correspond to the spatial positions of sarcomeric Z-disks; the distance between two neighboring Z-disks is interpreted as an individual SL. (B) The calculated individual SLs as a function of their X-positions in the striation profile obtained from raw or algorithmically adjusted data. (C) The distributions of SL obtained from raw or algorithmically adjusted data. All data are shown for an individual exemplary cardiac cell.

The analysis of variability in individual sarcomere lengths was implemented by custom-made software EqapAll6 (IDE Delphi Embarcadero). Full-frame transmitted light images were taken in “unattached”, “attached”, and “stretched” states, each with five ROIs to cover as much area of a cell as possible. The lengths of individual sarcomeres were determined using an algorithm based on the analysis of the sarcomere striation profile in each ROI as input data (Fig. 2A). In brief, the algorithm works as follows: 1) input data are analyzed for the presence of non-periodic changes in intensity (typically due to the uneven brightness or image artifacts) which were then removed using low-frequency filtering, 2) the corrected input data were next filtered by moving summing up of the values and the resulting signal is leveled and scaled according to the initial range of intensity (Fig. 2A, bottom part of the panel), 3) next, the adjusted data were analyzed for the presence of local maxima by finding the spatial position of sign reversion of the first derivative of the signal, 4) when that position was found, the local maximum was calculated by parabolic approximation of three neighboring positions of the signal, where the central position is at the point of sign reversion of the first derivative. The X-axis positions of local maxima were then interpreted as Z-disks and were used for calculation of the individual SL as a distance between two neighboring Z-disks (Fig. 2B). The use of a moving sum to smooth the striation profile allowed for more accurate determination of the local maxima by rejecting false, noise- or artifact-related, local peaks; this approach also resulted in much denser SL distribution (Fig. 2C). In addition, the calculated SL values from all 5 ROIs were combined into a single individual SL data set and only values between 1.2 and 2.6 μm were used in further analysis of SL variability.

### Statistical analysis

Statistical analyses were carried out with GraphPad Prism 7.0 (GraphPad Software). To verify that the samples of individual SL data do or do not follow a Gaussian distribution, both D’Agostino-Pearson and Shapiro-Wilk normality tests were applied. Thus, these normality tests were applied to the mean/median SL values and the values of corresponding SL variability measures, all treated as samples. The evaluation of significance in the mean/median SL values as well as the absolute/relative measures of SL variability between “unattached”, “attached” and “stretched” states was implemented by the Friedman test with Dunn’s multiple comparisons test that is appropriate for samples with a non-normal shape of distribution. Differences were considered significant at *p* < 0.05. Data are presented as mean ± S.D.

## RESULTS

We evaluated whether stretch itself can affect SL variability in the non-stimulated fully relaxed cardiomyocyte in the following states: without CF attachment (“unattached”), with CF attachment but without stretch (“attached”), and with stretch by CF (“stretched”). Only cells measured in all three states were used for the analysis.

First, we analyzed how many sets of individual SL values followed a normal distribution and whether this was affected by CF attachment or stretch. Out of total 36 analyzed SL data sets (12 sets per each state), 7 sets (19%) and 6 sets (17%) were non-normal according to the Shapiro-Wilk and D’Agostino & Pearson normality tests, respectively. The majority of SL data sets that did not pass these normality tests were obtained for “unattached” or “attached” states (11 sets), compared to the “stretched” state (2 sets).

Next, we analyzed quantitative statistical measures calculated for the individual SL data sets – mean, median, standard deviation, median absolute deviation, etc… – obtained from all cells (*n* = 12) for normality of their distribution. It was needed for proper selection of significance comparison test between each two states of the cells. Most of the statistical measures passed both D’Agostino & Pearson and Shapiro-Wilk normality tests but the following did not: inter-quartile range (IRQ), median absolute deviation (MAD), IQR-to-median SL value, and MAD-to-median SL value. We therefore used the Friedman test with Dunn’s multiple comparisons test to compare each statistical measure between two states of a cell, *i.e*. “unattached” *vs* “attached”, “unattached” *vs* “stretched”, “attached” *vs* “stretched”, assuming non-normal distribution of the samples.

**Figure 3:**
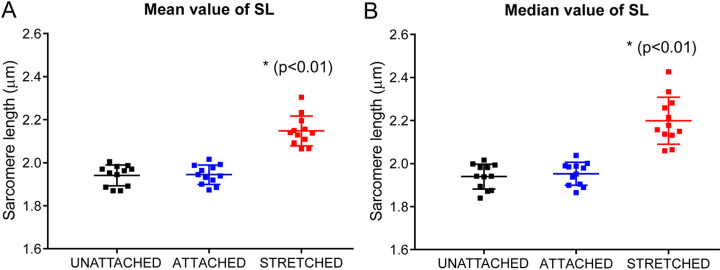
Stretch of resting cardiomyocytes affects sarcomere length (SL) mean and median values. The carbon fiber (CF) technique was used to test each myocyte in three states: without CF attachment (“unattached”), with CF attached but without stretch (“attached”), and with ~10% cell stretch applied (“stretched”). Measurements were made in transmitted light mode for *n* = 12 isolated cardiomyocytes from *N* = 7 hearts. (A) Mean SL value, and (B) Median SL value. * Difference between “stretched” state and either “unattached” or “attached” state is significant at *p*< 0.01 (Friedman’s test with Dunn’s multiple comparisons test).

The mean SL values were as follows: 1.94 ± 0.05 μm in the “unattached” state, 1.94 ± 0.04 μm in the “attached” state, and 2.15 ± 0.07 μm in the “stretched” state. A significant difference in mean SL was found for “stretched” state *vs* either “unattached” or “attached” state (*p* = 0.0001 and p = 0.0033, respectively), but not between the two latter states (Fig. 3A). Likewise, median SL values were found to be 1.94 ± 0.06 μm in the “unattached” state, 1.95 ± 0.05 μm in the “attached” state, and 2.20 ± 0.11 μm in “stretched” state, with a significant difference between “stretched” state *vs* either “unattached” or “attached” state (*p* = 0.0007 for both comparisons) but not between the two latter states (Fig. 3B).

**Figure 4:**
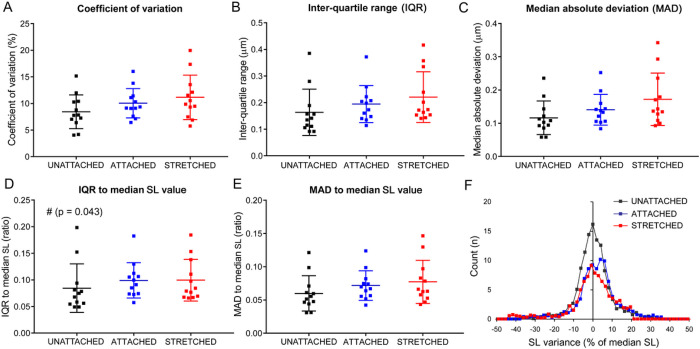
Stretch of resting cardiomyocyte does not affect the variability in individual sarcomere lengths (SL). The measurements were obtained in transmitted light mode for *n* = 12 isolated cardiomyocytes (each in “unattached”, “attached”, and “stretched” state) from *N* = 7 hearts. The following absolute and relative quantitative measures of SL variability were analyzed: (A) Coefficient of variation, (B) Inter-quartile range (IQR), (C) Median absolute deviation (MAD), (D) IQR divided by median SL value, and (E) MAD divided by median SL value. No significant differences were observed between “unattached”, “attached” and “stretched” states (except for the inter-quartile range divided by median SL value between “unattached” *vs* “attached”, indicated by “#” on panel D). (F) Average distribution plots for individual SL values obtained in “unattached”, “attached”, and “stretched” states. The peak of the plots corresponds to the averaged median SL value and the distributions are shown as a % of the corresponding median SL value.

**Figure 5:**
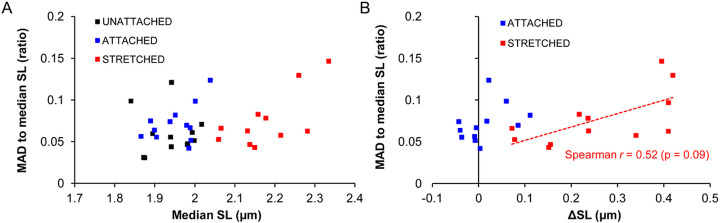
Lack of significant relationship between averaged sarcomere length (SL) and extent of SL variability. The measurements were obtained in transmitted light mode for *n* = 12 isolated cardiomyocytes (each in “unattached”, “attached”, and “stretched” state) from *N* = 7 hearts. (A) MAD-to-median SL value is plotted as function of average SL for all three states. (B) MAD-to-median SL value is plotted as function of absolute change in average SL (ΔSL) for “attached” and “stretched” states (ΔSL are calculated relatively to “unattached” state). Note that there is no significant relationship between ΔSL and the extent of SL variability (Spearman *r*= 0.52 with *p* = 0.09).

An important finding in non-stimulated fully relaxed isolated cardiomyocytes, imaged by the TL mode, was that the extent of SL variability, as evaluated by several absolute and relative indexes, was neither affected by the CF attachment itself, nor by cell stretch (Fig. 4A–E). This could also be demonstrated by direct comparison of SL distribution plots obtained for these states of the cells, where the width of the plots remains virtually identical regardless the state (Fig. 4F). Also, if we plotted the measure of SL variability (for example, MAD-to-median SL value) against the median SL value itself for all three states, we did not see a functional relationship between the groups of data (Fig. 5A). Finally, we calculated delta values between SL obtained in “unattached” and “attached”/”stretched” states, with further plotting of these delta SL values *vs* MAD-to-median SL values (Fig. 5B). The relationship for “attached” state was uncertain because the CF attachment did not produce any effect on the average SL. In the “stretched” state, however, there was also non significant relationship between the extent of SL variability and average SL value (e.g. Spearman *r* value was 0.52, *p* = 0.09). These findings indicate that SL variability is not sensitive to average sarcomere length *per se*.

## DISCUSSION

In the present study, we tested the effect of increase in average SL, i.e. the direct effect of preload, on the variability in the individual SLs in rat ventricular cardiomyocytes held in a fully relaxed state. We found that neither the extent of SL variability nor the character of this variability is affected by the preload itself. Therefore, experimentally observed increase in the SL heterogeneity in activated cardiomyocytes [17, 21] or in skeletal muscle [4, 16, 25, 27] appears to be related to the inhomogeneous process, both spatially and in time, of Ca^2+^ activation of individual sarcomeres, but not to the actual change in average SL.

However, our finding was obtained in fully relaxed cardiomyocytes, i.e. with low Ca^2+^ and BDM uncoupling between actin filament and myosin motors – a situation that may not be present under physiological conditions. Under normal conditions, a quiescent myocyte still experiences a certain level of background interaction between actin and myosin due to the non-disturbed myosin activity and a low but physiologically important diastolic level of [Ca^2+^]_i_ [9, 22]. This may predetermine an initial extent of SL non-uniformity in a resting (but not fully relaxed) cardiomyocyte. Accordingly, in an activated cell the contractile response of individual sarcomeres may be substantially non-synchronous [18, 37], thereby contributing to increased SL inhomogeneity due to the in-series mechanical communication between strongly activated (contracting) and weakly activated individual sarcomeres that are lengthened by their contracting neighbors [13].

The phenomenon of SL non-uniformity has already been extensively studied in skeletal muscle [4, 11, 33]. SL heterogeneity is substantially increased even if only a single sarcomere is disrupted in the initially untreated myofibril [12] and this is accompanied by diminished myofibrillar active force production [23]. Importantly, SL heterogeneity is even more increased in a muscle that is subjected to mechanical stretch upon activation [29, 30, 31] – these results clearly indicates the importance of dynamic redistribution of individual SLs in activated muscle. In contrast to the multitude of studies in skeletal muscle, the direct effect of sarcomere pre-stretch on SL variability in contracting cardiac myocytes remains to be determined. To elucidate this aspect in detail, future studies on SL variability should include cardiomyocytes that start their contraction from different end-diastolic SL (i.e. varied preloads).

A limitation of the present study is the use of non-confocal transmission light (TL) mode of measurements. The images taken in this mode are less accurate in terms of spatial determination of sarcomeres [17, 36]. An important note has been raised in [32], where the authors experimentally verified that the nanometer variations in SL, when obtained by conventional light microscopy, should not be interpreted as a real variation but rather as noise. They suggested reasonable and optimal levels of optical resolution – 100 and 50 nm, respectively – that are necessary to conform to a reliable quantification of SL variability. The use of advanced optical methods such as second-harmonic generation microscopy [10] may help to satisfy the requirements imposed by the recently introduced term “SL nanometry” [34, 35, 38]. Nevertheless, we recently reported hat SL variability measured by confocal microscopy in fluorescently stained (ANEPPS) non-stretched fully relaxed isolated rat cardiomyocytes was nearly the same by magnitude as for the TL mode in the current study (Fig. 2F in [20]). Similar results were also obtained in another study that combined confocal and TL modes of SL measurements [28]. Of note, our recent and current studies employed comparable spatial resolutions of the optical systems. We therefore conclude that, provided the optical settings provide proper spatial resolution, the TL imaging may be at least as good for SL analysis as confocal imaging.

## Data Availability

Data acquired and analyzed during this study are available from the corresponding author upon reasonable request.
